# The Histone Lysine Demethylase JMJD3/KDM6B Is Recruited to p53 Bound Promoters and Enhancer Elements in a p53 Dependent Manner

**DOI:** 10.1371/journal.pone.0096545

**Published:** 2014-05-05

**Authors:** Kristine Williams, Jesper Christensen, Juri Rappsilber, Anders Lærke Nielsen, Jens Vilstrup Johansen, Kristian Helin

**Affiliations:** 1 Biotech Research and Innovation Centre (BRIC), University of Copenhagen, Copenhagen, Denmark; 2 Centre for Epigenetics, University of Copenhagen, Copenhagen, Denmark; 3 Wellcome Trust Centre for Cell Biology, University of Edinburgh, Edinburgh, United Kingdom; 4 Biopharmaceutical Research Unit, Novo Nordisk A/S, Måløv, Denmark; 5 The Danish Stem Cell Center (DanStem), University of Copenhagen, Copenhagen, Denmark; Peking University Health Science Center, China

## Abstract

The JmjC domain-containing protein JMJD3/KDM6B catalyses the demethylation of H3K27me3 and H3K27me2. JMJD3 appears to be highly regulated at the transcriptional level and is upregulated in response to diverse stimuli such as differentiation inducers and stress signals. Accordingly, JMJD3 has been linked to the regulation of different biological processes such as differentiation of embryonic stem cells, inflammatory responses in macrophages, and induction of cellular senescence via regulation of the *INK4A-ARF* locus. Here we show here that JMJD3 interacts with the tumour suppressor protein p53. We find that the interaction is dependent on the p53 tetramerization domain. Following DNA damage, JMJD3 is transcriptionally upregulated and by performing genome-wide mapping of JMJD3, we demonstrate that it binds genes involved in basic cellular processes, as well as genes regulating cell cycle, response to stress and apoptosis. Moreover, we find that JMJD3 binding sites show significant overlap with p53 bound promoters and enhancer elements. The binding of JMJD3 to p53 target sites is increased in response to DNA damage, and we demonstrate that the recruitment of JMJD3 to these sites is dependent on p53 expression. Therefore, we propose a model in which JMJD3 is recruited to p53 responsive elements via its interaction with p53 and speculate that JMJD3 could act as a fail-safe mechanism to remove low levels of H3K27me3 and H3K27me2 to allow for efficient acetylation of H3K27.

## Introduction

The N-terminal tails of histone proteins are subject to various post-translational modifications including methylation of lysine residues. The combination of histone modifications affects chromatin structure and can determine transcriptional outcome. In addition, histone modifications have been implicated in the regulation of genomic stability and cell fate decisions, as well as pathological processes such as cancer development.

Di- and tri-methylation of histone 3 lysine 27 (H3K27me2/me3) is catalysed by the Polycomb Repressive Complex 2 (PRC2), and is associated with transcriptional repression. The Polycomb group (PcG) proteins are essential for normal development in *Drosophila* and mammals, and are found as key regulators of genes involved in cellular differentiation and stem cell identity [1]–[4]. In addition, PcG proteins can repress the expression of certain tumour suppressor genes, including the *INK4A-ARF* locus [5]–[8] and overexpression of PcG proteins has been implicated in cancer development [9], [10].

The JmjC domain containing proteins JMJD3/KDM6B and UTX/KDM6A are H3K27me2/me3 specific demethylases [11]–[15]. With the ability to revert PcG mediated repression, the proteins are potential mediators of differentiation and development. In agreement with this, the *C. elegans* UTX and JMJD3 homologs are required for normal gonadal development in the worm [11], [16] and inhibition of Utx1 expression in zebrafish results in improper posterior development [14]. Unlike UTX, JMJD3 appears to be highly regulated at the transcriptional level and is upregulated in response to diverse stimuli such as differentiation inducers and stress signals. For instance, JMJD3 is dynamically expressed during differentiation of embryonic stem cells [17] and keratinocytes [18], and is highly upregulated in inflammatory stimulated bone marrow-derived macrophages [12], [19]. Furthermore, JMJD3 possesses tumour suppressor characteristics and is upregulated in response to oncogenic stress, where it contributes to activation of the *INK4A-ARF* locus [20], [21].

*Trp53*, the p53 tumour suppressor gene, is frequently mutated in human cancer. In unstressed cells, p53 levels are kept low by the negative regulator MDM2, which is an E3 ubiquitin ligase that binds and targets p53 for proteosomal degradation [22]. However, in response to stress signals, such as DNA damage, p53 is subject to post-translational modifications including phosphorylation, which reduces its affinity for MDM2 leading to stabilization of the protein. Release of p53 from MDM2 allows it to function as a transcription factor, where it binds to DNA as a tetramer leading to activation of multiple target genes involved in processes like cell cycle arrest, apoptosis, senescence and DNA repair [23]. Most cancer derived p53 mutations are found within the DNA binding domain [24], suggesting that the main tumour suppressing role of p53 is based on its ability to function as a transcription factor. However, it is now evident that p53 signalling is mediated at several levels and that it also has cytoplasmic roles, where it can function in the regulation of apoptosis and autophagy [25].

The exact mechanism by which p53 discriminates between different cell fates in response to numerous types of stress is still an open question. There appears to be a complex network of signalling pathways involved in the regulation of p53 protein stability and p53 signalling pathways. p53 is subject to various post-translational modifications including phosphorylation, ubiquitination and methylation, and it has been reported to associate with several different binding partners [26], which could all contribute to selectively target p53 to certain genes.

Consistent with recent reports [27]–[29] we show here that JMJD3 interacts with p53. By performing genome-wide mapping of JMJD3 and p53, we find that JMJD3 binding shows significant overlap with p53 targeted sites. We find that the binding of JMJD3 to p53 bound promoters, as well as p53 associated enhancer elements, is increased in response to DNA damage and demonstrate that the recruitment of JMJD3 to these sites is dependent on p53.

## Results

### JMJD3 associates with p53

**Figure 1:**
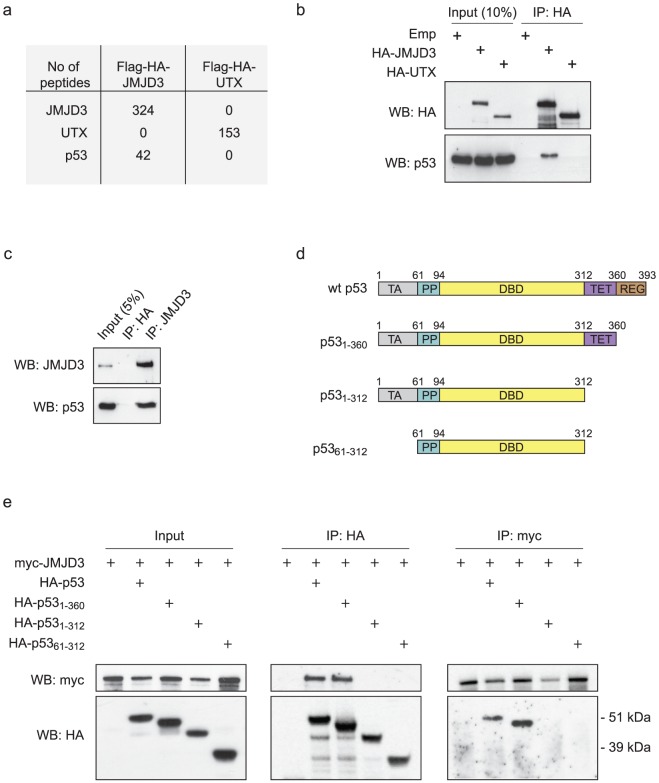
JMJD3 interacts with p53. a, The number of JMJD3, UTX and p53 peptides identified by mass spectrometry in the tandem anti-Flag-HA purifications of Flag–HA–JMJD3 and Flag-HA-UTX stably expressed in HEK293 cells. **b**, Phoenix cells were transfected with HA-JMJD3, HA-UTX or an empty vector (Emp), and immunoprecipitated with an antibody against the HA-tag. Western blotting was performed with antibodies against HA or p53. **c**, Co-immunoprecipitation of endogenous JMJD3 and p53 was performed in Phoenix cells by immunoprecipitating with HA (negative control) or JMJD3 antibody. **d**, Schematic representation of wild type (wt) p53 and the three p53 deletion mutants lacking the regulatory (REG), the tetramerization (TET) or the transactivating (TA) domains as indicated. **e**, Pheonix cells were transfected with myc-tagged JMJD3 alone or together with wt p53 or p53 deletion mutants as indicated. Immunoprecipitation was performed with myc- or HA antibody followed by western blotting.

To gain information regarding the functional role of JMJD3, we purified JMJD3-associated proteins by tandem affinity purification. We expressed double-epitope (Flag-HA) tagged JMJD3 or UTX in HEK293 cells, and purified interaction partners from nuclear extracts by Flag- followed by HA-affinity purification (Fig. S1a). Co-purified proteins were subsequently analysed by mass spectrometry. From this analysis, we identified p53 as an interaction partner of JMJD3, but not UTX (Fig.1a). Instead, UTX co-purified with several members of the MLL3/4 complex as previously reported [15], [30], [31] (Fig. S1b). To verify the interaction between JMJD3 and p53, we transfected Phoenix cells with plasmids expressing HA-tagged JMJD3 or HA-tagged UTX, and were able to co-immunoprecipitate (co-IP) p53 only when overexpressing JMJD3 (Fig. 1b). Moreover, the interaction was validated between endogenous JMJD3 and p53 in Phoenix cells (Fig. 1c).

p53 has a domain structure similar to other transcription factors and possesses an N-terminal transactivating domain, a proline rich region, a central DNA-binding domain, a tetramerization domain and a C-terminal regulatory domain. To map the region of p53 required for binding to JMJD3, we tested a number of p53 mutant proteins (Fig. 1d): p53_1–360_ lacking the C-terminal regulatory domain; p53_1–312_ lacking the regulatory and the tetramerization domains; and p53_61–312_ lacking the regulatory, the tetramerization and the transactivation domains. As shown in Fig. 1e, the mutants of p53 lacking the tetramerization domain do not interact with JMJD3. These data demonstrate an interaction between JMJD3 and p53 that is likely to be mediated either directly through the p53 tetramerization domain, or that tetramerization of p53 is required for efficient binding of JMJD3.

### Genome-wide mapping of JMJD3 and p53 binding sites in IR treated BJ cells

**Figure 2:**
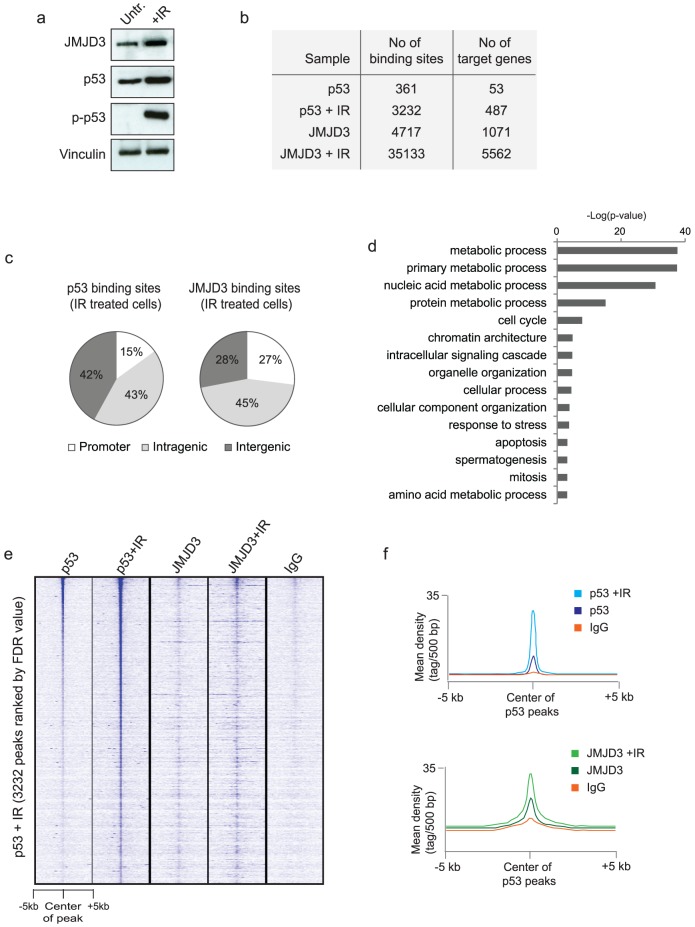
Genome-wide binding of JMJD3 and p53 before and after IR treatment. a, Western blot of JMJD3, p53, p-p53 (p53 serine 15 phosphorylation) and vinculin in human immortalized BJ fibroblasts either untreated or 6 hours after exposure to IR. **b**, The number of identified binding sites and target genes for p53 and JMJD3 (defined as binding of p53 or JMJD3 +/− 5 kb from TSS) in untreated or IR exposed cells. **c**, The distribution of identified p53 peaks (left panel) or JMJD3 peaks (right panel) in IR treated cells into promoter regions (+/−5 kb from TSS), intragenic regions or intergenic regions (> 5 kb from a gene). **d**, Gene Ontology analysis of JMJD3 target genes in IR treated BJ cells. **e**, Heat map of p53 and JMJD3 ChIP-seq data from untreated or radiated cell representing the 3232 identified p53 binding sites. The heat map is ranked according to the FDR-value of the p53 peaks. **f**, The average distribution of p53, JMJD3 and IgG across the centre of all p53 peaks before and after IR.

Our results indicate that JMJD3 specifically associates with the tetrameric form of p53, which is the active DNA-binding form of p53. To understand if JMJD3 and p53 also display genomic co-localization, we performed global mapping of JMJD3 and p53 binding sites in telomerase-immortalized human BJ diploid fibroblasts by chromatin immunoprecipitation followed by sequencing (ChIP-seq). The experiments were performed in untreated cells or five hours following exposure to ionizing radiation (IR). IR generates DNA double-stranded breaks, which is a potent inducer of the DNA damage response. Following exposure to IR, p53 was upregulated and phosphorylated on serine 15 (Fig. 2a), which indicates efficient activation of p53 and release from MDM2. In addition, JMJD3 protein levels were also increased in response to IR (Fig. 2a), which is consistent with our previous results, in which we demonstrated upregulation of JMJD3 in response to UV-induced DNA damage [20]. We also confirmed here that JMJD3 is upregulated in response to UV damage (Fig S2a), and verify that the observed interaction between JMJD3 and p53 still occurs after exposure to either IR or UV damage (Fig S2b and c).

From the ChIP-seq experiments we found that both p53 and JMJD3 were bound to significantly more DNA sites following IR compared to untreated cells (Fig. 2b). Analysis of the ChIP-seq data by peak detection identified 3232 genomic sites bound by p53 in radiated BJ cells and 35133 JMJD3 binding sites (Fig. 2b), corresponding to 487 and 5562 unique genes targeted by p53 or JMJD3 (+/− 5 kb from TSS), respectively (Fig. 2b). Besides binding to promoters of its target genes, p53 often binds to response elements several kb upstream of genes or in exon/intron regions [32], [33]. In agreement with this, we found that only 15% of p53 binding sites were located in promoter regions of annotated genes (+/− 5 kb from TSS) (Fig. 2c). For JMJD3, we found that 27% of the binding sites were associated with a promoter (Fig. 2c). At these genes, JMJD3 displayed the strongest binding at the TSS, with some spreading of the signal into the gene body (Fig. S3a and S3b). Using available data on the distribution of H3K4me3 and H3K27me3 in BJ cells [34], [35], we found that JMJD3 targeted promoters were strongly associated with the activating H3K4me3 mark and depleted for H3K27me3 (Fig. S3a and S3c) corresponding to the reported enzymatic activity of JMJD3 and in agreement with previously reported genome-wide binding data of JMJD3 obtained in LPS-induced macrophages [19]. Gene ontology analysis of the identified JMJD3 target genes showed an enrichment of genes involved in a variety of basic cellular processes (Fig. 2d), including various metabolic pathways. However, interestingly, the analysis also identified a subset of JMJD3 target genes involved in regulation of cell cycle, response to stress and apoptosis. These are all well-known p53 regulated processes, and consistently, we found that these cellular processes were also enriched in the identified p53 target genes (Fig. S4). In order to systematically investigate if JMJD3 exhibits a DNA damage responsive recruitment to p53 binding sites, we generated a heat map representing the 3232 identified p53 binding sites (Fig. 2e). This analysis demonstrated an enrichment of p53 at these binding sites in response to IR (Fig. 2e and Fig. 2f, upper panel). Interestingly, we also detected an overall recruitment of JMJD3 to these target sites in response to IR (Fig. 2e and Fig. 2f, lower panel).

Taken together, the genome-wide data on p53 and JMJD3 binding demonstrate that both proteins are upregulated and recruited to DNA sites in response to DNA damage. Although JMJD3 is bound to more genomic sites than p53, we find that a subset of JMJD3 target genes is involved in the regulation of cell cycle and apoptosis. In agreement with this, we observe an overall recruitment of JMJD3 to p53 binding sites in response to DNA damage.

### JMJD3 and p53 co-localize at promoters and distal regulatory elements

**Figure 3:**
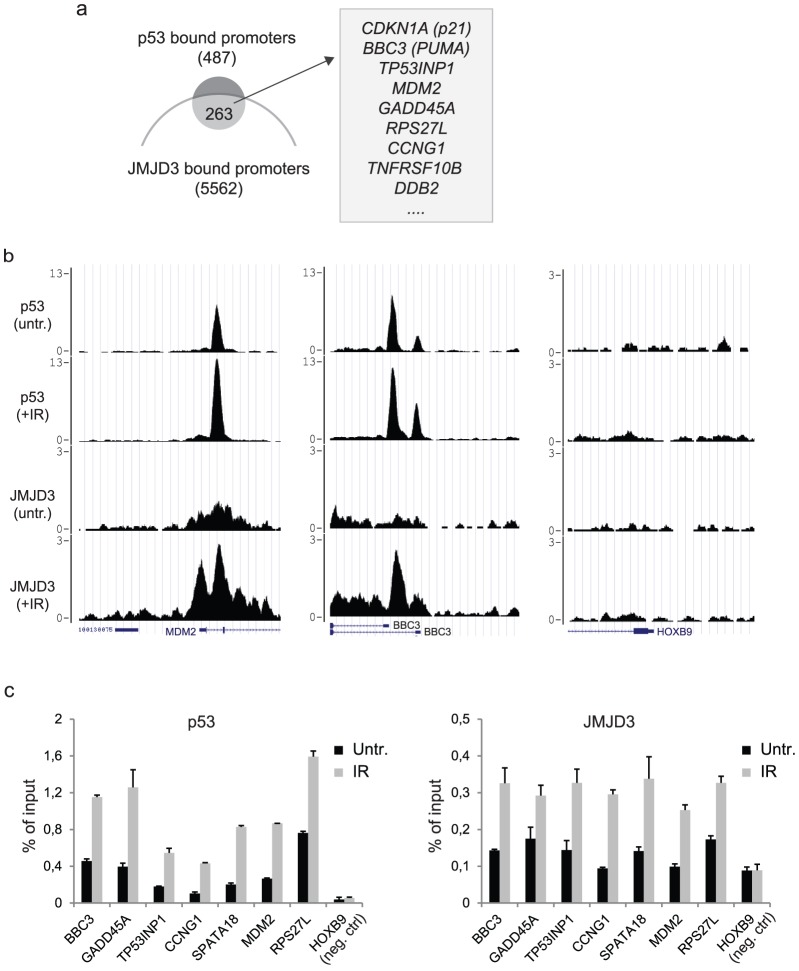
IR responsive recruitment of JMJD3 and p53 to promoter regions. a, Venn diagram demonstrating significant (p<10^−8^) overlap between JMJD3 and p53 target genes in the IR treated BJ cells. At least 263 genes were found to be bound by both JMJD3 and p53 in the promoter regions, which included several well-known p53 target genes such as *CDKN1A, BBC3, TP53INP1, MDM2, GADD45A, RPS27L, CCNG1*, *TNFRSF10B,* and *DDB2.* **b**, Examples of p53 (before and after IR treatment) and JMJD3 (before and after IR treatment) ChIP-seq tracks at *BBC3*, *MDM2, TP53INP1*, *RPS27L* and *HOXB9* (negative control). y-axis of binding profiles denotes number of sequence tag reads. **c**, The corresponding p53 and JMJD3 ChIP-qPCR validations at the TSS of the genes listed above.

After detecting that a fraction of JMJD3 is associated with p53 binding sites, we focused on p53 binding sites that were located in gene promoter regions. By overlaying the identified JMJD3 and p53 promoter-bound genes, we detected a significant overlap of target genes in irradiated BJ cells (Fig. 3a). In this analysis, 263 p53 target genes were co-bound by JMJD3, which gives a highly significant overlap between JMJD3 and p53 target genes (p<10^−8^). These included most of the best-characterised p53 target genes involved in cell cycle regulation (e.g. *CDKN1A (p21), CCNG1*), DNA repair (e.g. *GADD45A*, *DDB2*) apoptosis (*e.g. BBC3 (PUMA), TNFRSF10B, TP53INP1*) and p53 regulation (*e.g. MDM2*) (Fig. 3a). We verified that p53 and JMJD3 are co-recruited to promoters of several target genes, illustrated by ChIP-seq binding profiles (Fig. 3b) and independent ChIP-qPCR validations (Fig. 3c). Furthermore, we found that H3K4me3 is enriched and H3K27me3 is depleted on JMJD3 and p53 targeted promoters (Fig. S5). Whereas we observed a slight increase of H3K4me3 levels in response to IR, we did not detect a corresponding decrease in the levels of H3K27 methylation (Fig. S5). This is consistent with the observation that both JMJD3 and p53 appear to be bound (although at lower levels) to several of their common target genes in the absence of DNA damage (Fig. 2e and f).

In addition to binding promoter regions of target genes, we found that the majority of p53 binding sites are distributed in inter- and intragenic regions (Fig 2c), indicating that p53 largely localizes at distal enhancer elements in response to IR. Enhancers are regulatory DNA elements often located far away from the genes they act upon. They are characterized by binding of multiple transcription factors that subsequently recruit co-activator proteins such as the histone acetyltransferase p300 [36]. Moreover, the presence of transcription factors at enhancers is associated with low occupancy of nucleosomes and they are therefore sensitive to DNase treatment [37], [38]. Genome-wide studies have also demonstrated that enhancers can be annotated based on the existence of specific histone modifications. Whereas enhancers are generally depleted for H3K4me3, they are instead associated with H3K4me1 [39]. Moreover active enhancers are characterized by high levels of H3K27ac, whereas inactive (transcriptionally poised) enhancers have increased levels of H3K27me3 [40]–[42].

**Figure 4:**
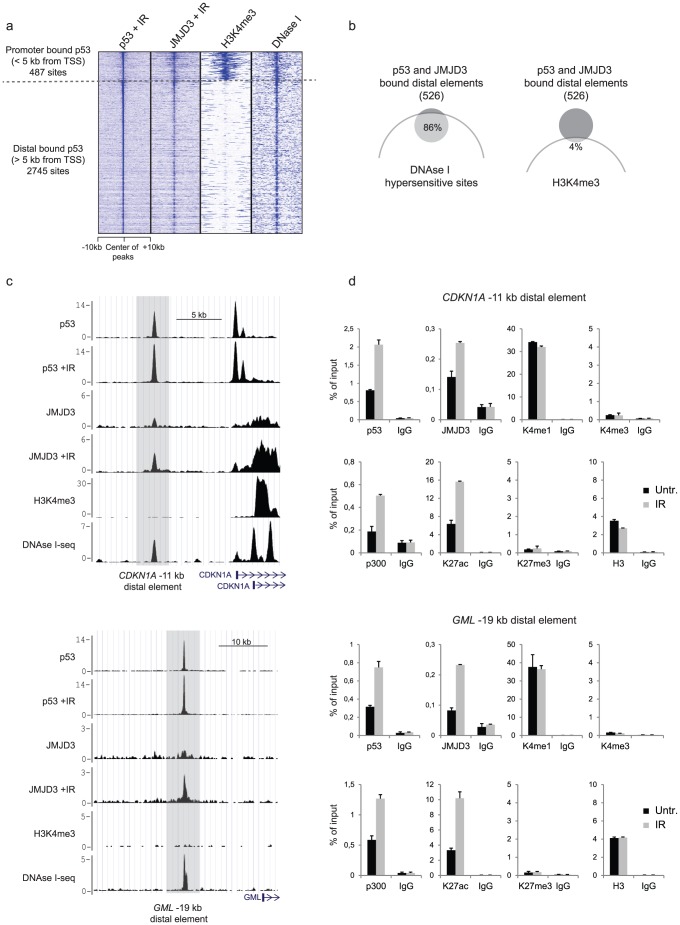
IR responsive recruitment of JMJD3 and p53 to distal enhancer elements. a, Heat map representing p53 binding sites divided into promoter associated (< 5 kb from a TSS, 487 peaks) or distal binding sites (> 5 kb from a TSS, 2745 peaks). **b**, Venn diagrams illustrating the overlap between p53 and JMJD3 co-bound distal elements and DNase I hypersensitive sites (left panel) or H3K4me3 positive regions (right panel). **c**, Examples of p53 (before and after IR treatment) and JMJD3 (before and after IR treatment), H3K4me3 and DNase I-seq [34], [35], [43] tracks at two putative enhancer elements located 11 kb upstream of *CDKN1A* (upper panel) or 19 kb upstream of *GML* (lower panel). **d**, Corresponding ChIP-qPCR validations of the binding of p53, JMJD3 and p300 as well as the levels of histone modifications H3K4me1, H3K4me3, H3K27ac and H3K27me3 at the two distal binding sites listed above.

To investigate the genomic co-localization of JMJD3 and p53 at distal p53 binding elements, we divided all p53 binding sites into promoter-associated sites (< 5 kb from a TSS) or distal p53 binding sites (> 5 kb from a TSS) (Fig. 4a). As shown in Figure 4a, Jmjd3 is enriched at p53-bound distal elements (Fig. 4a). In fact, 526 distal DNA elements were significantly enriched by both p53 and JMJD3. By including genome-wide data on DNase I hypersensitivity and H3K4me3 from BJ cells [34], [35], [43] we observed that while p53 bound promoters were both DNase I hypersensitive and associated with H3K4me3, the p53 bound distal elements were largely depleted from H3K4me3 but still DNase I hypersensitive (Fig. 4a). Thus, we found that 86% of the distal DNA elements, which were co-bound by JMJD3 and p53, overlap with a DNase I hypersensitive site. On the other hand, only 4% are positive for H3K4me3 (Fig. 4b), indicating that these sites are likely to represent regulatory enhancer elements. Furthermore, we validated by ChIP-qPCR that p53 and JMJD3 display an IR responsive recruitment to several putative enhancer elements (Fig.4c and d and Fig. S6a and S6b). We confirmed that these sites possess features of active enhancers; they are DNase I positive (Fig. 4c), show binding of p300 and have enrichment of H3K4me1 and H3K27ac but not H3K4me3 and H3K27me3 (Fig. 4d). We also observed that exposure to IR correlated with an increased binding of p300 and subsequently acetylation of H3K27, which is in agreement with data demonstrating that p53 interacts with p300 [44].

In summary, these results demonstrate that JMJD3 co-localizes with p53 at promoters, as well as distal enhancer elements, and confirms that the binding of p53 and JMJD3 to these sites increases after exposure to IR.

### JMJD3 recruitment to p53 binding sites is dependent on p53

**Figure 5:**
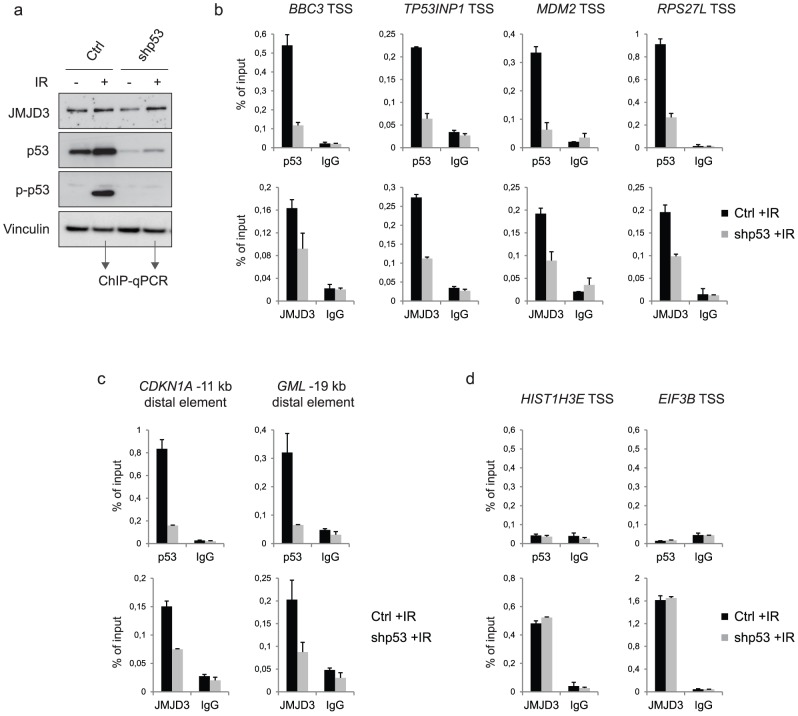
JMJD3 binding to p53 binding sites is dependent on p53. a, Western blot of control (ctrl) or p53 knockdown (shp53) cells. The cells were exposed to IR and harvested for ChIP-qPCR assays after five hours. **b and c**, ChIP-qPCR data demonstrating the binding of p53 and JMJD3 to the promoters of p53 target genes *BBC3*, *TP53INP1, MDM2,* and *RPS27L* (b) or to the *CDKN1A* -11 kb and *GML* -19 kb distal elements (c) in control or p53 knockdown cells. **d**, ChIP-qPCR data demonstrating the binding of p53 and JMJD3 to *HIST1H3E* and *EIF3B*, which are not p53 target genes.

Although JMJD3 associates with chromatin it does not contain a known DNA binding domain. Therefore, it is assumed that JMJD3 depends on other proteins for the specific recruitment to genomic target sites. p53, on the other hand, contains a DNA binding domain that specifically recognizes a DNA element known as the p53 response element. Since we found an increase of JMJD3 binding to p53 genomic binding sites in response to DNA damage, we investigated if p53 is involved in the recruitment of JMJD3 to a subset of its binding sites. Therefore, we knocked down p53 in BJ cells using short hairpin RNA (shRNA) targeting p53 (Fig. 5a). Both control and p53 knockdown cells were exposed to IR, and harvested for ChIP assays five hours later. As expected, we observed significantly lower binding of p53 at targeted promoters (Fig. 5b) and distal enhancer elements (Fig. 5c) in the p53 depleted BJ cells compared to control cells. However, we also observed decreased JMJD3 binding to these sites in the p53 knockdown cells, demonstrating that p53 is required for efficient binding of JMJD3 to both promoter regions and distal enhancer elements. Importantly, we found that JMJD3 enrichment at target genes that were not bound by p53, were unaffected by depletion of p53 (Fig. 5d), indicating that p53 depletion does not affect the overall chromatin binding ability of JMJD3, but is involved in the direct tethering of JMJD3 to sites where the two proteins co-localise.

### Potential function JMJD3 binding to p53 target sites

To understand a potential functional role of the observed recruitment of JMJD3 to p53 genomic targets, we tested different potential regulatory mechanisms of JMJD3. One possibility is that JMJD3 contributes to the regulation of p53 methylation, as previously suggested using an antibody raised to methylated lysine [29], but not confirmed in an independent study [28]. The C-terminal part of p53 is methylated at lysine residues K370, K372, and K382 [45]–[47] and was demonstrated to be demethylated by the histone lysine demethylase LSD1 at K370me2 [48]. To test if JMJD3 can demethylate p53 on any of these sites, we purified recombinant catalytically active JMJD3 (JMJD3s, amino acids 1027–1685) (Fig. S7a and S7b) [11]. Next we incubated recombinant JMJD3s with H3K27me3 synthetic peptide (amino acids 20–34) or p53 peptides (amino acids 367–388) with the following modifications: unmodified, K370me2, K372me1, K372me2, K372me3, K373me2, K381me1, K381me2, K381me3, K382me1, K382me2, K382me3 or K386me2, and analyzed the samples by MALDI-TOF mass spectrometry. Whereas, JMJD3 efficiently catalysed demethylation of the H3K27me3 peptide (Fig. S8a), no detectable activity was observed with any of the tested p53 peptides (Fig. S8b). These results disfavour a role for JMJD3 in the regulation of p53 C-terminal methylation, however, we cannot rule out that JMJD3 is involved in regulating p53 methylation at other lysine residues than the tested ones, or that additional co-factors are required for an efficient enzymatic removal of methylation.

As previously mentioned, we were unable to detect a decrease in H3K27me3 levels after recruitment of JMJD3 to p53 target sites (Fig. S5). Also, when estimating the overall levels of H3K27me3 at p53 targeted promoters and enhancers, we found that these were generally depleted of H3K27me3 (Fig. S9). These data indicate that JMJD3 is not involved in demethylating high levels of H3K27me3 at p53 binding sites. Moreover, we also tested if depleting JMJD3 in the BJ cells had an effect on the transcription of p53 target genes. However, a 50–70% knockdown of JMJD3 (mRNA and protein levels, respectively) (Fig. S10a and S10b) did not cause any detectable changes of the expression of selected p53 target genes after DNA damage (Fig. S10c). Furthermore, JMJD3 knockdown did not affect the protein levels of p53 (Fig S2a and S10a). Taken together, these data suggest that JMJD3, under these conditions, is not a major regulator of p53 transcriptional activation of target genes or p53 protein stability.

**Figure 6:**
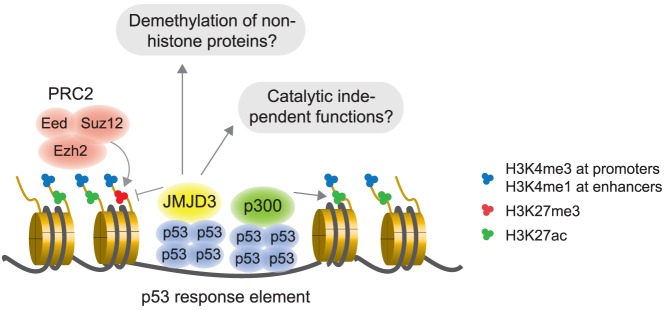
Model of the potential roles of JMJD3 in regulating p53 target genes. JMJD3 is recruited to p53 bound promoters and distal enhancer elements via an interaction with the p53 tetramer, which simultaneously recruits the histone acetyltransferase p300. JMJD3 could be involved in adjusting p53 transcriptional regulation by demethylating H3K27me3/me2, demethylating other non-histone proteins, or by having catalytically independent functions. See text for further details.

Based on our data, we propose a model where JMJD3 is recruited to p53 bound promoters and distal enhancer elements via an interaction with the p53 tetramer, which simultaneously recruits the histone acetyltransferase p300 (Fig. 6). We speculate that JMJD3 could serve as a fail-safe mechanism to remove low levels of slowly accumulating H3K27me3 or H3K27me2. However, we cannot rule out that that JMJD3 could have histone demethylase independent roles such as in the recruitment of other effector proteins or in the demethylation of non-histone targets (Fig. 6).

## Discussion

The expression of the H3K27me3/me2 demethylase JMJD3 is increased in response to different stimuli such as differentiation inducers and stress signals. It has been linked to the regulation of numerous biological processes including embryonic stem cell differentiation, inflammatory response in macrophages and regulation of the *INK4A-ARF* locus during stress. With its tissue specific and highly inducible expression, JMJD3 appears to function in well-defined and restricted cellular processes, which is unlike UTX that is ubiquitously expressed and suggested to function as a “housekeeping” demethylase. However, little is known about the direct function of JMJD3 in transcriptional regulation. Here we show that JMJD3 interacts with the tumour suppressor protein p53, and that JMJD3 localises to p53 bound promoters and enhancers in a p53-dependent manner.

By purifying JMJD3 and UTX associated proteins, we identified p53 as an interaction partner of JMJD3, which is consistent with recent studies [27]–[29]. For UTX, on the other hand, we did not observe an interaction with p53, but instead purified several members of the MLL3/4 complex. This is in agreement with previously reported data [15], [30], [31]. We did not find significant enrichment of MLL proteins in the JMJD3 complex purification, indicating that UTX and JMJD3 associate with distinct protein complexes.

We further characterised the interaction between JMJD3 and p53 by cloning different p53 deletion mutants. We found that the tetramerization domain of p53 is required for the interaction between p53 and JMJD3. Moreover, we performed genome-wide mapping of JMJD3 and p53 by ChIP-seq in telomerase immortalized BJ fibroblasts after exposure to IR, which induces DNA damage and p53 activation. Here we found that JMJD3 associates with genes involved in basic cellular processes, but also genes involved in cell cycle regulation, stress responses and apoptosis. In agreement with this, we observed a significant overlap of JMJD3 and p53 target genes, which included several well-characterised p53 responsive genes. In addition, we found that JMJD3 co-localizes with p53 at its distal binding sites, which are likely to represent enhancer elements. We found that JMJD3 is upregulated in response to DNA damage and that the binding of JMJD3 to both p53 targeted promoters and enhancers increases after IR treatment. Furthermore, we were able to demonstrate that the binding of JMJD3 to p53 binding sites is dependent on p53, and thereby we provide a potential recruitment mechanism for JMJD3 to a subset of its target genes.

We also tested if JMJD3 could be involved in demethylating p53. p53 is methylated at C-terminal lysine residues: K370, K372 and K382. Interestingly, the enzymes responsible for p53 methylation, SET9 [45], SMYD2 [46] and SET8 [47], are enzymes that also target histone tails. In line with this, the histone lysine demethylase LSD1 was reported to demethylate p53 K370me2 [48]. We therefore tested the ability of JMJD3 to demethylate p53 C-terminal peptides that were mono-, di- or tri-methylated at several lysine residues. However, whereas we found a clear demethylation of H3K27me3 peptide, we did not detect demethylation of any of the tested p53 peptides. These data suggest that JMJD3 is not involved in demethylating the C-terminal part p53. However, there could still be undiscovered methylation sites in the p53 protein that are targeted by JMJD3 demethylase activity. Alternatively, JMJD3 could be involved in demethylating other non-histone proteins localised at p53 target genes.

Based on our observations, we propose a model where JMJD3 binds to p53 responsive elements via its association with the p53 tetramer. We did not find a correlating decrease in H3K27me3 after the increased binding of JMJD3 to p53 target genes. This is consistent with data reported by De Santa et al., who investigated the recruitment of JMJD3 to target genes in inflammatory stimulated macrophages and did not find any correlation between JMJD3 recruitment and changes in H3K27me3 levels [19]. Furthermore, JMJD3 has been shown to have demethylase independent roles in chromatin remodelling [49] and in its negative regulation of reprogramming of somatic cells into induced pluripotent stem cells [50]. Taken together, these results suggest a demethylase independent role of JMJD3 similar to what has been described for other demethylases, including UTX [51], [52].

In a recent study in which the role of p53 during differentiation and DNA damage of human embryonic stem cells is investigated[27], the authors found genes associated with p53 during differentiation to show a corresponding decrease in H3K27me3 levels. These results suggest that, p53 target genes, under certain circumstances, could be regulated by H3K27me3. Moreover, these observations could give mechanistically insight to the observed interaction between p53 and JMJD3. The lack of H3K27me3 demethylation after recruitment of JMJD3 to p53 target genes in our study could be explained by the fact that we often observe that both p53 and JMJD3 are bound to their target genes even before DNA damage. Therefore, we speculate that JMJD3 could acts as a fail-safe mechanism to maintain the levels of H3K27me3 low and permissible for H3K27ac by p300 at p53 response elements, rather than contributing to the direct activation of target genes. This mechanism could be especially important at enhancer regions, which has been suggested to be regulated by the switch between H3K27 methylation and acetylation. Interestingly, a few reports have linked JMJD3 to the regulation of enhancer activity. For example, JMJD3 was suggested to regulate H3K27me3 levels at an enhancer element driving the expression of the *BCL2* gene in breast cancer cells [53]. Another study demonstrated the requirement of JMJD3 recruitment to an enhancer element regulating α-globin expression, in order to mediate PRC2 eviction and loss of H3K27me3 [54].

Within the last decade, the regulatory functions of p53 have expanded from cell cycle arrest and apoptosis to also include metabolism, aging, embryo implantation and quiescence of stem cells [55], [56]. It is therefore likely that the importance of JMJD3 in p53 transcriptional regulation would be more pronounced when investigating other p53 regulated pathways than the IR induced DNA damage response, as was the case in our study. Indeed, understanding the molecular mechanisms involved in p53 mediated transcriptional regulation is of great interest in order to gain insight into the important tumour suppressive functions of p53, as well as its expanding roles in metabolism, cellular homeostasis and differentiation. Hopefully, future studies will help unravel the exact role of JMJD3 in the complex network of p53 transcriptional signalling.

## Materials and Methods

### Cell culture and DNA damage

A telomerase-immortalized version of the human diploid BJ fibroblast cell line (ATCC number CRL-4001), HEK293 cells [57], and Phoenix cells [58] were cultured in D-MEM (Gibco) supplemented with 10% FBS (Hyclone), penicillin and streptomycin. Recombinant lentiviruses encoding p53 shRNA [59] were produced by standard methods employing transfection of pRetroSuper shRNA in Phoenix-ampho cells. shRNA-transduced BJ cells were selected 36 h post transduction with 2 µg per ml of puromycin for 72 h. To generate DNA damage, BJ cells were exposed to 10 Gy of ionizing radiation delivered by X-ray generators (Faxitron CP160, 160 kV, 6.3mA and Faxitron RX650, 130 kV, 3 mA).

### Cloning Procedures

JMJD3 and UTX were cloned as previously described [11]. Wt p53 and p53 deletion mutants were PCR-amplified from human p53 clone [60] and introduced into the Gateway Entry vector pCR8/GW/TOPO (Invitrogen) following the manufacturer's protocol. The different constructs were subcloned in the desired vectors by Gateway technology (Invitrogen).

### Purification of JMJD3 and UTX complexes

In order to isolate JMJD3 and UTX-containing complexes, two-step affinity purification was performed followed by mass spectrometry analysis. HEK293 cells stably expressing doxycycline (DOX)-inducible amino-terminally Flag and HA-tagged JMJD3 or UTX were generated using the HEK293 Flp-In TREx cells as described by the manufacturer (Invitrogen). Nuclear extracts (250–500 mg, 3×10^9^ cells) from the HEK293 cell lines were precleared and incubated with a 700 µl packed volume of anti-Flag-beads (Anti-Flag M2-agarose, Sigma) overnight at 4 °C with rotation. The beads were collected by centrifugation at 700 × g for 5 min and washed 6 times with 40 × resin bed volume of buffer A (20mM Tris-HCl, pH 8.0, 300 mM NaCl, 1.5 mM MgCl_2_, 0.2 mM EDTA, 10% glycerol, 0.2 mM PMSF, 1 mM DTT, 1 µg/ml aprotinin and 1 µg/ml leupeptin). The beads were transferred into a 10 ml poly-prep chromatography column (Bio-Rad) and complexes were then eluted five times after 10 min of incubation using one resin bed volume of buffer A supplemented with 0.5 µg/μl Flag peptide. The eluate was subjected to a second round of purification using an antibody against the HA-tag. The Flag-IP elute was incubated with 200 µl of a 50% slurry of HA-beads overnight. The beads were washed four times with buffer A and eluted with 100 µl buffer A supplemented with 1 µg/μl HA peptide for two hours. Small aliquots of the eluted proteins were run on SDS-PAGE gels and subjected to silver staining using the SilverQuest kit (Invitrogen) according to manufacturer's instructions. The rest of the samples were boiled in SDS loading buffer and run shortly into a SDS-PAGE gel in order to remove the Flag and HA peptide and other contaminations. A gel slice containing the purified proteins was isolated for mass spectrometry analysis.

### Antibodies

The JMJD3 antibody was generated in rabbits, using affinity-purified GST-JMJD3 (amino acids 798–1095) as described in [20]. Other antibodies used included p53 (DO-1), p-p53 (Cell Signaling, #9284), p300 (Santa Cruz, Sc-585) H3 (Abcam, Ab1791), H3K4me1 (Abcam, Ab8895), H3K4me3 (Cell Signaling, #9751), H3K27ac (Milipore, 07-360), H3K27me3 (Cell Signaling, #9733) and Vinculin (Sigma-Aldrich, V9131).

### Co-immunoprecipitation experiments and WB analysis

Co-mmunoprecipitation experiments were performed using 1 mg of cell extracts in E1A buffer (50 mM Hepes buffer pH 7.5, 250 mM NaCl, 0, 5 mM EDTA, 0.1% TritonX100, Leupeptin, Aprotinin and 1 mM PMSF) as described earlier [61] using the indicated antibodies. Protein extracts for western blot of p53 and JMJD3 were made using RIPA buffer (25 mM Tris-HCl pH 7.6, 150 mM NaCl, 1% NP-40, 1% sodium deoxycholate, 0.1% SDS, Leupeptin, Aprotinin and 1 mM PMSF) and sonication. Immunoblotting was performed according to standard protocols.

### ChIP and ChIP-seq

ChIP-seq experiments were performed as previously described [61]. ChIP-seq data are available at the Gene Expression Omnibus (GEO) (accession number GSE55912).Tags were aligned to the human genome (hg18 assembly) using Bowtie version 0.12.7 [62] with default parameters except “-S -m 1”. Peak detection for JMJD3 and p53 datasets was performed in MACS version 2 [63], using sequencing reads from an IgG control experiment as negative control. When generating bigwig files we allowed only one read per chromosomal position thus eliminating potential spurious spikes. Each remaining read was extended from its 5′-end to a total length of 250 bases. Each bigwig file was also scaled to TPM (Tags Per Million) based on the number of unique read positions. Heat map and binding profiles across genomic regions were generated using the SeqMiner program [64], where a constant read number between samples was employed for comparison. Gene Ontology classifications were performed using the Panther program [65]. Primers for ChIP-qPCR analyses were designed using the Primer3 software and real-time quantitative PCR was performed on a Roche LightCycler 480 II detection system using SYBR Green qPCR Master Mix (Fermentas).

### Purification of recombinant JMJD3

Recombinant JMJD3 was generated by co-transfection of baculovirus transfer-vector containing a Flag-tagged fragment of human JMJD3 (JMJD3s, amino acids 1027-1685) and Bsu36I linearized Bakpak6 baculovirus DNA. Insect cells were incubated at 28°C and harvested 40–44 h post infection, washed twice in PBS, resuspended in 30 ml of lysis buffer (25 mM HEPES-NaOH pH 7.5, 300 mM NaCl, 1.5 mM MgCl2, 10% Glycerol, 0.2% TritonX100, Leupeptin, Aproteinin), sonicated and cleared by centrifugation at 20,000 g for 30 min. After filtrating the lysate through a 0.45 µM filter, it was loaded onto a 10 ml poly-prep chromatography column (Bio-Rad) packed with 0.5 ml packed volume of Flag-beads. The column was washed with 2×10 ml of lysis buffer and eluted in buffer supplemented with 0.5 µg/μl Flag peptide. The eluted fractions were analysed by SDS-PAGE, flash frozen in liquid N_2_ and stored at −80°C.

### Demethylation assay

Demethylation assays were performed on calf thymus histones (Sigma Aldrich) or synthetic peptides H3K27me3 (amino acids 20-34: LATKAARKSAPATGG) or p53 (amino acids 367–388: SHLKSKKGQSTSRHKKLMFKTE) with the following modifications: unmodified, K370me2, K372me1, K372me2, K372me3, K373me2, K381me1, K381me2, K381me3, K382me1, K382me2, K382me3 or K386me2. 15 µg of histones or 4 µg of peptides were incubated with purified Flag-JMJD3s for 60 min at 37°C in demethylation buffer (25 mM Tris pH8, 1.5 mM MagCl_2_, 50 mM NaCl, 1 mM α-Ketoglutaric acid, 2 mM Ascorbic acid, 40 µM FeSO_4_) in a final volume of 100 µl. For analysis of histones increasing amount of recombinant Flag-JMJD3s (0–30 µg) were used and reaction mixtures were analysed by western blotting using specific antibodies against H3K27me2, H3K27me3, or H3K4me3. For peptide analysis 30 µg of recombinant Flag-JMJD3s were added. After incubation in demethylation buffer the peptide samples were added 1/10 volume 1% TFA, and desalted on a μC18 ZipTips (Millipore) following the suppliers instructions. Peptides were eluted in a saturated solution of α-cyano-4-hydroxycinnamic acid in 65% acetonitrile, in Milli-Q water containing 0.1% TFA. 1 µl was spotted onto the target and air-dried for 15 min at rt. The MALDI-TOF MS was carried out on an Ultraflex TOF/TOF (Bruker) operated in positive ion mode with an ion source voltage of 25 kV, a lens voltage of 7.5 kV and a reflector voltage of 26.3 kV. The system was run in deflection mode with a mass suppression of 500 Da. The data analysis was carried out using the FlexAnalysis software (Bruker). Baseline subtraction and smoothing of the curves were applied.

## Supporting Information

Figure S1 **Purification of Flag-HA-JMJD3 and Flag-HA-UTX complexes**. a, Silver-stained SDS-PAGE of Flag- and Flag-HA-purified complexes from HEK293 cells stably expressing Flag-HA-JMJD3 or Flag-HA-UTX. The arrows indicate the position of Flag-HA-JMJD3 or Flag-HA-UTX. **b**, The number of peptides identified by mass spectrometry for JMJD3, UTX, MLL4, MLL3, RBBP5, WDR5, ASH2L, PTIP, DPY-30 and p53 in the Flag-HA tandem purifications.
(EPS)

Figure S2 **JMJD3 is upregulated and interacts with p53 after UV damage.** a, Western blot of JMJD3, p53, and vinculin in human immortalized BJ fibroblasts either untreated or 6, 12 or 24 hours after exposure to UV. b, Co-immunoprecipitation of endogenous JMJD3 and p53 was performed in Phoenix cells by immunoprecipitating with HA (negative control) or JMJD3 antibody before or after 5 h of IR (10 Gy). c, Co-immunoprecipitation of endogenous JMJD3 and p53 performed in Phoenix cells by immunoprecipitating with HA (negative control) or p53 antibody before or after 5 h of UV (50 J/m^2^).
(EPS)

Figure S3 **Characterization of JMJD3 target genes.** a, Heat map of JMJD3 (before and after IR treatment), H3K4me3 and H3K27me3 [34], [35] at all identified JMJD3 target genes in IR treated BJ cells. **b**, Average binding profile of JMJD3 across the TSS of its target genes. **c**, Average distribution of H3K4me3 and H3K27me3 at JMJD3 target genes.
(EPS)

Figure S4 **Gene Ontology analysis of identified p53 target genes.** Gene Ontology analysis of the p53 target genes identified in the IR treated BJ cells (defined as genes bound by p53 +/- 5 kb from TSS).
(EPS)

Figure S5 **Investigation of histone modifications at p53 bound promoters.** ChIP-qPCR assays estimating the levels of H3, H3K4me3, and H3K27me3 at p53 target genes *BBC3*, *RPS27L, CDKN1A, CCNG1, MDM2, SPATA18, TP53INP1,* and the repressed *HOXB9* gene that is not bound by p53 or JMJD3.
(EPS)

Figure S6 **IR responsive recruitment of JMJD3 and p53 to distal enhancer elements.** a, Examples of p53 (before and after IR treatment) and JMJD3 (before and after IR treatment), H3K4me3 and DNase I-seq [34], [35], [43] tracks at two putative enhancer elements located 12 kb upstream of *ATF3* (upper panel) or at genomic position *chr4∶153,399,193-153,401,046* (lower panel). **b**, Corresponding ChIP-qPCR validations of the binding of p53, JMJD3 and p300 as well as the levels of histone modifications H3K4me1, H3K4me3, H3K27ac and H3K27me3 at the two distal binding sites listed above.
(EPS)

Figure S7 **Purification of recombinant catalytically active JMJD3.** a, Samples of purified recombinant JMJD3 (JMJD3s, amino acids 1027-1685) were subjected to SDS-PAGE and stained using Coomassie blue. The arrow indicates the position of recombinant JMJD3. **b**, The activity of recombinant JMJD3 was tested *in vitro* by incubating histones with increasing amounts of recombinant JMJD3 in demethylase buffer. The samples were analysed by western blotting for H3K27me3, H3K27me2 and H3K4me3.
(EPS)

Figure S8 **Demethylation of H3K27me3 or p53 peptides.** a and b, H3(20-34)K27me3 (a) or p53 (unmodified, K370me2, K372me1, K372me2, K372me3, K373me2, K381me1, K381me2, K381me3, K382me1, K382me2, K382me3 or K386me2) (b) peptides were incubated with or without recombinant JMJD3 and analysed by mass spectrometry. A shift in mass equivalent to one methyl-group is indicated by dashed lines.
(EPS)

Figure S9 **p53 binding does not correlate with H3K27me3.** a, Heat map of p53 and H3K27me3 [34], [35] at p53 bound promoters (< 5kb from TSS) or p53 distal binding sites (> 5kb from TSS) in IR treated BJ cells. **b,** Average distribution of H3K27me3 and p53 across p53 promoter associated or distal binding sites.
(EPS)

Figure S10 **Expression of p53 target genes after JMJD3 knockdown.** a, Western blot of JMJD3, p53, and vinculin in human immortalized BJ fibroblasts transfected with a control siRNA (siScr) or a siRNA targeting JMJD3 (siJMJD3). **b** and **c**, qRT-PCR data illustrating the expression of *JMJD3* (b), *CDKN1A*, *BBC3* and *GADD45A* (c) in control or JMJD3 knockdown cells before or 6 and 24 h after IR treatment.
(EPS)
